# General practice consultation patterns and patient factors predicting older patients’ use of out-of-hours services: a nationwide register-based cohort study

**DOI:** 10.3399/BJGP.2024.0798

**Published:** 2025-05-15

**Authors:** Jonas Olsen, Sonja Wehberg, Frans Boch Waldorff, Daniel Pilsgaard Henriksen, Jesper Lykkegaard

**Affiliations:** 1 Research Unit for General Practice, Department of Public Health, University of Southern Denmark, Odense, Denmark, 5230; 2 Section of General Practice and The Research Unit for General Practice, Department of Public Health, University of Copenhagen, Copenhagen, Denmark; 3 Department of Clinical Pharmacology, Odense University Hospital, Odense, Denmark

**Keywords:** accident and emergency medicine, aged, health services accessibility, health services for the aged, primary health care

## Abstract

**Background:**

Out-of-hours primary care services cannot provide the same continuity and coordination of care as daytime general practice. Thus, patients with high risk of complex care trajectories should, when possible, be seen by the GP during daytime opening hours. However, little is known about how provision of daytime consultation services affects older patients’ out-of-hours healthcare seeking.

**Aim:**

To analyse how patient characteristics and general practices’ patterns of daytime consultations relates to their older patients’ use of out-of-hours services.

**Design and setting:**

This was a register-based cohort study of all Danish citizens aged ≥75 years in the Northern, Central, Southern, and Zealand Region of Denmark in 2017–2021.

**Method:**

The general practices’ frequencies of daytime consultations were adjusted for patient population characteristics. Latent profile analysis identified group patterns of daytime consultation types and frequencies. Zero-inflated Poisson regression was used to analyse how patient characteristics and practice pattern of daytime consultations were associated with older patients’ use of out-of-hours primary care.

**Results:**

Increasing age, multimorbidity, number of drugs, level of home healthcare services, and non-Western ethnicity were associated with being a user of out-of-hours services and higher frequency of consultations. Older patients in the general practices that provided substantially more daytime consultations than most general practices had no fewer consultations in out-of-hours primary care services.

**Conclusion:**

Use of out-of-hours primary care services depends more on patient characteristics than general practice organisation of daytime consultations. The findings suggest that increasing the number of daytime consultations may not reduce use of out-of-hours services.

## How this fits in

This study provides critical insights into the dynamics between daytime general practice consultations and the use of out-of-hours primary care services among older patients. It highlights that patient characteristics, such as age, multimorbidity, medication use, ethnicity, and home healthcare needs, are more significant determinants of out-of-hours service use than the frequency of daytime consultations provided by general practices. The findings suggest that increasing the number of daytime consultations may not reduce use of out-of-hours services. The conclusions underscore the importance of understanding patient demographics when designing effective primary care strategies.

## Introduction

Out-of-hours primary care services (OOH) are designed to manage acute conditions that cannot wait until the following workday.^1^ Consultations in OOH are more expensive than in daytime general practice^2^ and OOH cannot provide the same continuity and coordination of care as the GP.^1^ Furthermore, older patients are more often admitted to the hospital when treated by an unfamiliar doctor^3–5^ likely owing to high levels of multimorbidity and risk of complex care trajectories.^6^ Consequently, OOH should only be used in case of urgency and not because of lack of convenient access to the GP, as studies have shown to occur.^7,8^

In a study, by the same author group, four distinct patterns of how general practices organise their daytime consultations for older patients were identified.^9^ The majority profile of practices (77–92%) provided roughly 5.0 face-to-face, 3.5 telephone, 2.2 email consultations, and 0.4 home visits and chronic care reviews each year per older patient, and there were three secondary profiles which were practices that all provided more consultations than the 'Majority': with twice the telephone and 1.3 the total consultations ('Phone heavy' 9–11%); with 1.1 the face-to-face, 1.4 the telephone, twice the email, and 1.3 the total consultations ('High frequency' 11–14%); and with 1.4 the telephone, 2.2 the email, and 1.5 the total consultations ('Phone and email heavy' 8%).^9^

The practice profiles present an opportunity to study if older patients use OOH less when listed with general practices with higher frequency of consultations and to what extent the composition of consultation types matters. There are two hypotheses for how more consultations in daytime general practice might reduce OOH use: (a) by ensuring access to timely primary care during acute illness^10^ and (b) by long-term prevention and treatment of risk factors and progressive diseases.^11–13^ The aim of this study was to analyse how patient characteristics and general practices’ patterns of daytime consultations relates to older patients’ use of OOH.

## Method

### Design, setting and population

A nationwide register-based cohort study was conducted following a published protocol.^14^

The Northwestern European country of Denmark has approximately 5.8 million citizens. Most healthcare services are fully tax paid, including daytime general practice and OOH. More than 98% of the population is registered with a self-chosen general practice. Older patients are on average registered with the same general practice for at least 9.5 years.^15^ The general practices must serve their listed patients from 8 am to 4 pm on all workdays (Monday to Friday).^2^ Throughout the study the OOH in the included regions were organised as GP cooperatives (GPC) available only out-of-hours. First contact is always by telephone to a GP, 59% of all contacts are concluded by telephone consultation with 2.6% as direct hospital admissions.^16^

The GPs electronically report each performed service to the regional health insurance for remuneration. The available services in OOH are face-to-face, telephone, and video consultations, and home visits (Supplementary Box S1). Video consultations were introduced in 2020.

For each of the years 2017–2020, the study included all people aged ≥75 years in the Northern, Central, Southern, and Zealand Region of Denmark who on their birthday in the given year were registered with a Danish general practice. All general practices in the four regions were included. Practices with <500 registered, or <20 eligible patients, at the beginning or end of each study year and follow-up year were excluded for the current year. Practices without ≥1 renumerated consultation with one patient each month of the study and follow-up year were excluded for the current year. The patients were followed until death, migration, or change of general practice.

### Outcomes and covariates

Data sources and linkage between sources are described in Supplementary Box S2.

Patient characteristics were assessed at 1 January for each study year. Included characteristics were age, sex, multimorbidity (Nordic Multimorbidity Index^17^), ethnicity, number of drugs, level of home healthcare services, rural or urban district, travel distance from home address to the general practice and to the local emergency department, cohabitation, household income, and household wealth (Supplementary Box S3). If missing, patient distance data were imputed as the median within the municipality. Missing ethnicity and household income and wealth was categorised as missing. Patients lost to follow-up were included with their time until lost to follow-up.

The general practices’ pattern of daytime consultations was defined by latent profile analysis of frequency of daytime face-to-face, telephone, email, and home visit consultations, and chronic care reviews adjusted for the abovementioned patient characteristics (Supplementary Box S4).^18^

In the current study the authors first analysed the association between patient characteristics and OOH use. Next, the relationship between the general practice’s pattern of organisation and OOH, adjusted for patient characteristics, was analysed.

**Figure 1.**
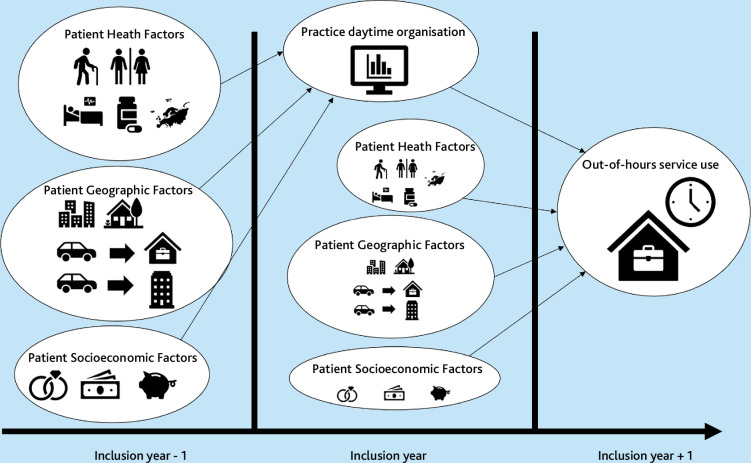
Overall study design. Note that modelling of practice daytime organisation is from Olsen *et al*.^18^

The main outcome was overall frequency of OOH consultations and secondary outcomes were the frequencies of the individual consultation types in the following year (Figure 1). Both daytime consultations and OOH use were adjusted for patient factors to account for temporal data separation and to limit confounding. Contacts on the same date were merged following the hierarchy home visit > face-to-face > video > telephone.

Association between patient characteristics and the general practices pattern of daytime consultations and use of OOH was calculated by zero-inflated Poisson regression. The model allows for simultaneous estimation of the odds ratio (OR) for being a user of OOH (OR<1) or non-user (OR>1), that is, patients with or without consultations in OOH, and estimation of the incidence rate ratio (IRR) for yearly contacts if a patient is a user of OOH. A sensitivity analysis was performed investigating OOH use within the same year as the general practices’ latent profile classification (Supplementary Figure S1).

All statistical analyses were performed using Stata 18.0.

### Patient and public involvement

A panel of older patients and next of kin assisted with hypothesis generation before analysis. A panel of GPs assisted with interpretation of the study results.

## Results

In 2017, the study included 295 583 patients listed with 1016 general practices, and in 2020, 344 008 patients listed with 981 general practices. The average age of the patients at baseline was 80 years and 151 888 (44.2%) were males. The median number of prescriptions drugs was 4, 34 684 (10.1%) received municipality home healthcare service, and 14236 (4.1%) were nursing home residents (Table 1). Distance data were missing for 1% of patients in 2017 and 10% in 2021.

**Table 1.** Baseline practice and patient factors

Practice factors	2017	2018	2019	2020
Practices, *n*	1016	1001	992	981
Patients ≥75 years, median (IQR)	241 (156–382)	258 (163–401)	278 (176–429)	295 (182–458)
Number of GPs, median (IQR)	2 (1–3)	2 (1–3)	2 (1–3)	2 (1–3)
Age of GPs, years, mean	53.6	53.1	52.9	52.9
Seniority of GPs, (years), mean	14.8	14.6	14.5	14.6
**Patient health factors**				
Patients, *n*	295 583	307 458	324 701	344 008
Age, years, mean	80.3	80.2	80.1	80.0
Male sex, %	127 680 (43.2)	133 691 (43.5)	142 515 (43.9)	142 515 (43.9)
Nordic Multimorbidity Index, median (IQR)	3 (0–10)	3 (0–10)	3 (0–10)	3 (0–10)
Ethnic Danish, %	286 586 (97.0)	297 922 (96.9)	314 546 (96.9)	333 298 (96.9)
Number of prescription drugs, median, (IQR)	4 (2–7)	4 (2–7)	4 (2–7)	4 (2–7)
Receiving home healthcare services, %	44 170 (14.9)	42 983 (14.0)	43 171 (13.3)	34 684 (10.1)
Nursing home residents, %	13 024 (4.4)	12 912 (4.2)	13 710 (4.2)	14 236 (4.1)
**Patient geographic factors**				
Region, (%)				
Northern	44 486 (15.1)	46 575 (15.1)	49 099 (15.1)	51 806 (15.1)
Central	89 704 (30.3)	91 289 (29.7)	97 890 (30.1)	105 055 (30.5)
Southern	98 803 (33.4)	101 493 (33.0)	105 665 (32.5)	111 338 (32.4)
Zealand	62 590 (21.2)	68 101 (22.1)	72 047 (22.2)	75 809 (22.0)
Rural district, (%)	30 570 (10.3)	32 567 (10.6)	35 582 (11.0)	38 215 (11.1)
Travel distance to practice, km, median (IQR)	3 (1–6)	3 (1–7)	3 (1–7)	3 (1–7)
Travel distance to hospital, km, median (IQR)	20 (7–31)	19 (7–31)	19 (7–31)	19 (7–31)
**Patient socioeconomic factors**				
Cohabitating, %	146 760 (49.7)	155 413 (50.5)	166 585 (51.3)	172 766 (50.2)
Household income in EUR, median	24 045 (21 394–29 948)	24 731 (21 947–30 925)	25 354 (22 443–31 803)	26 093 (22 960–32 935)
Household wealth in EUR, median	72 651 (19 280–171 382)	77 196 (21 283–180 641)	80 171 (22 585–185 980)	84 923 (24 840–194 894)
**Years listed in the general practice, (%)**				
0–1	33 057 (11.2)	36 813 (12.0)	43 141 (13.3)	44 750 (13.0)
2–5	42 697 (14.4)	46 936 (15.3)	47 660 (14.7)	50 222 (14.6)
>5	219 829 (74.4)	223 709 (72.8)	233 900 (72.0)	249 036 (72.4)
Patients changing practice during follow-up, (%)	26 973 (9.8)	32 185 (11.1)	38 023 (12.5)	45 676 (9.9)

IQR = interquartile range.

The average number of OOH consultations per year decreased from 0.57 (SD 1.35) in 2018 to 0.48 (SD 1.21) in 2021. In 2021 the consultations comprised 0.29 telephone, 0.01 video, 0.06 face-to-face consultations, and 0.12 home visits. Telephone consultation stayed the same from 2018 to 2021 with a small increase in 2020 while face-to-face and home visits decreased in the period (Table 2).

**Table 2.** General practice pattern of daytime consultations and their patients use of out-of-hours in the following year^a^

		*n* (%)	Total	(SD)	Face-to-face	(SD)	Telephone	(SD)	Video	(SD)	Home visits	(SD)
**Year**	**Year and profile**											
**2017**	Overall	1016 (100.0)	0.57	(1.35)	0.08	(0.34)	0.28	(0.84)	n/a		0.20	(0.66)
	Majority	833 (82.0)	0.56	(1.36)	0.08	(0.33)	0.28	(0.85)	n/a		0.20	(0.66)
	High frequency	105 (10.3)	0.59	(1.36)	0.09	(0.35)	0.29	(0.81)	n/a		0.21	(0.69)
	Phone heavy	78 (7.7)	0.56	(1.31)	0.08	(0.34)	0.28	(0.79)	n/a		0.20	(0.67)
**2018**	Overall	1001 (100.0)	0.54	(1.34)	0.08	(0.35)	0.27	(0.83)	n/a		0.18	(0.63)
	Majority	820 (82.0)	0.54	(1.34)	0.08	(0.34)	0.27	(0.84)	n/a		0.18	(0.63)
	High frequency	116 (11.6)	0.55	(1.38)	0.09	(0.36)	0.28	(0.84)	n/a		0.18	(0.64)
	Phone heavy	65 (6.5)	0.52	(1.22)	0.08	(0.33)	0.27	(0.75)	n/a		0.17	(0.58)
**2019**	Overall	992 (100.0)	0.52	(1.32)	0.06	(0.28)	0.33	(0.99)	0.01	(0.07)	0.13	(0.48)
	Majority	909 (91.6)	0.52	(1.32)	0.06	(0.28)	0.33	(0.99)	0.01	(0.07)	0.12	(0.48)
	Phone heavy	83 (8.4)	0.52	(1.32)	0.06	(0.28)	0.33	(0.96)	0.01	(0.08)	0.14	(0.49)
**2020**	Overall	981 (100.0)	0.48	(1.21)	0.06	(0.29)	0.29	(0.84)	0.01	(0.09)	0.12	(0.49)
	Majority	914 (93.2)	0.48	(1.21)	0.06	(0.29)	0.29	(0.84)	0.01	(0.09)	0.12	(0.49)
	Phone and email heavy	67 (6.8)	0.47	(1.18)	0.06	(0.29)	0.29	(0.86)	0.01	(0.09)	0.11	(0.44)

^a^No video consultations in 2017–2019. SD = standard deviation.

### Patient factors associated with OOH use

**Figure 2.**
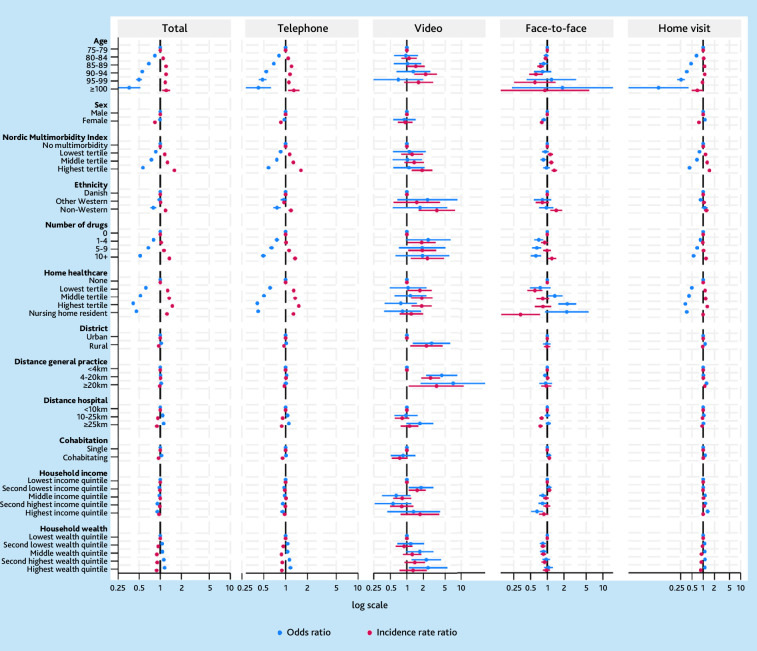
Zero-inflated Poisson regression for patient factors associated with consultations in out-of-hours services (2021). The figure displays the results of a zero-inflated Poisson regression for total consultations in out-of-hours services for older patients in 2021. Numbers >1 for IRR means more total consultations adjusted for all other parameters. Numbers <1 for OR means more likely to use out-of-hours services adjusted for all other parameters. Years refer to out-of-hours service use in the following year. IRR = incidence rate ratio. OR = odds ratio.

Higher patient age, multimorbidity, polypharmacy, home health care, and non-Western ethnicity was associated with being a user of OOH and more total consultations. Small to no differences were found between the user status of males and females. However, female sex was associated with fewer consultations. Increasing household income was associated with using OOH, however, with fewer consultations for the highest incomes. Living in a rural district was associated with fewer consultations. Increasing travel distance to the emergency department, cohabitation, and increasing household wealth was associated with non-use and fewer consultations. Small to no associations were found for travel distance between the patient and general practice (Figure 2). Overall, patterns for specific consultation types resembled that of total consultations. Estimations for video consultations had high levels of uncertainty. No major differences were observed from year to year (Supplementary Table S1a and S1b).

### Practice organisation and OOH use

In 2021, 249 036/344 008 (72%) of the patients had been listed with the same general practice for ≥5 years. During follow-up, 9–13% of the patients changed practice (Table 1). The 'Majority' profile comprised most general practices in each year (82–93%). The 'High frequency' profile was found in 2017 and 2018, the 'Phone Heavy' profile in 2017–2019, and a 'Phone and email heavy' profile in 2020 (Table 2).

No persistent pattern of association with use of or consultation frequency in OOH was found for the 'Phone heavy' or the 'High frequency' profiles compared with the 'Majority', although patients with practices in the 'High frequency' profile in 2017 were more likely to use the OOH in 2018 (OR 0.95 for non-use,95% confidence interval [CI] = 0.91 to 0.98) and patients with practices in the 'Phone heavy' profile in 2018 had fewer total OOH consultations in 2019 (IRR 0.97, 95% CI = 0.94 to 0.99).

**Figure 3.**
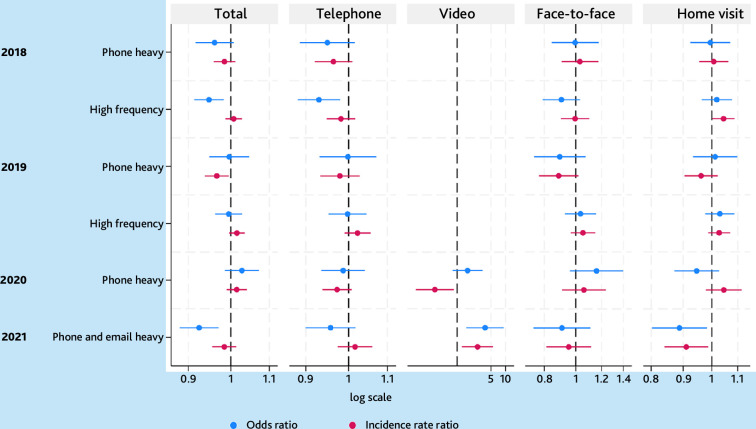
Zero-inflated Poisson regression for latent profiles of general practices’ pattern of daytime consultations associated with consultations in out-of-hours services (2018–2021). The figure displays the odds ratio, incidence rate ratio and confidence intervals of zero-inflated Poisson regressions for consultation types in out-of-hours services by latent profile of general practice pattern of daytime consultations. The 'Majority' profile was used as reference for each year. The models were adjusted for patient age, sex, multimorbidity, ethnicity, polypharmacy, level of home healthcare services, rural or urban district, travel distance from home address to the general practice and to the local emergency department, cohabitation, household income, and household wealth. Numbers >1 for IRR means more total consultations adjusted for all other parameters. Numbers <1 for OR means more likely to use out-of-hours services adjusted for all other parameters. Years refer to out-of-hours service use in the following year. IRR = incidence rate ratio. OR = odds ratio.

Patients with practices in the 'Phone and email heavy' profile identified in year 2020 were more likely to use OOH in 2021 (OR 0.92 for non-use, 95% CI = 0.88 to 0.97) and more likely to have received a home visit (OR 0.89 for non-use, 95% CI = 0.80 to 0.98). Estimations for video consultations had high levels of uncertainty. No association was found for face-to-face consultations nor for consultation frequency compared with the 'Majority' (Figure 3 and Supplementary Figure S1a and S1b).

The sensitivity analyses of OOH use in the same year as the latent profile classification showed no persistent pattern of association with use of or consultation frequency in OOH for the 'Phone heavy' or the 'High frequency' profiles compared with the 'Majority'. Patients with practices in the 'High frequency' profile were more likely to use OOH in 2017 and in 2018, they had more OOH consultations in 2018. In 2020, patients with practices in the 'Phone and email heavy' profile had more consultations in OOH compared with the 'Majority' (Supplementary Table S2).

## Discussion

### Summary

Age, number of drugs, level of home health care, and non-Western ethnicity had the strongest associations with using the OOH and frequency of consultations. Non-use was associated with increasing household wealth and increasing distance to the nearest emergency department. Older patients listed with general practices with higher daytime consultation frequency did not have fewer consultations in OOH.

### Strengths and limitations

The use of high-quality virtually complete nationwide registers reduced the risk of selection and information bias. Rich individual data on patients enabled adjustment for a plethora of health, geographic, and socioeconomic factors isolating the variation across practices not because of these factors, and limited risk of multicollinearity between variables as a result of the high power of the study.

Categorisation of daytime organisation as a whole comprised all the different consultation types is a strength compared with focusing on a single consultation type. Additionally, using existing groupings of general practices (latent profiles), and not theoretical or extreme cases improves the validity of this study's results and limits risks of skewing results because of potential outliers, which may have context-specific factors defining their outlier status.

General practices that changed pattern of daytime consultations during follow-up introduced undifferentiated variability in the models increasing the risk of a type II error — that is, a failure to reject the null hypothesis if it is in fact false. However, general practices, in general, stay within their profiles with a tendency to move towards the 'Majority' profile.^18^ The sensitivity analysis likewise strengthens the robustness of the results by reducing the temporal distance from exposure classification to outcome measurement. Although the analysis is limited by possible non-chronology between exposure and outcome, the consistent absence of association across both approaches supports the validity of the study's conclusions.

The study's modelling of general practices by daytime consultation patterns is limited by not distinguishing between acute effects (timely access) and long-term prevention. Likewise, different prioritisations within general practices might result in the same profile membership, although the underlying organisation differs.

As the data in the current study is based on the Danish population, some estimates (latent profiles) in the study's models may have limited external validity. However, as most of the findings on patient characteristics align with existing literature and given that the current study describes a general relationship between organisation of daytime general practice consultations and OOH use, the authors anticipate that the conclusions are generalisable to healthcare systems in Northern and Northwestern Europe with comparable general practice systems and who employ a GP-centred approach to OOH care.^1,19^

In this study's protocol a sensitivity analysis was described excluding the index patient from the calculation of their exposure status from the count of patients and services used to calculate the patient’s practice’s pattern of daytime consultations simulating a figuratively 'next patient' in the practice.^14^ However, following further reflection the authors decided against this. General practice adapts their services to all their patients, therefore there cannot exist a figuratively 'next patient' as the demands of this patient will affect the practice’s overall service. This holds true even for patients without demand in the practice (no consultations) owing to freeing up time for other patients.

A decision was taken to follow patients from the 1 January each year and not their birthday as this would require recalculation of all steps by the number of patients each year. In the case of high seasonality in mortality, emigration, or change of general practice, this might result in biased estimates, for example, if older patients who are frail more often die at the start of the year, they contribute less time compared with older patients who are not frail and the study's estimates might thus reflect a healthier population than the average. However, as it is anticipated that seasonality affects all general practices equally, the authors do not believe this had an impact on the study's conclusions.

The E-value and stratified analyses were not calculated owing to their being no need when only small to no associations were seen for the pattern of daytime consultations and OOH use.

The described variation analysis in the study protocol will be published in a separate paper for clarity of this manuscript.

### Comparison with existing literature

In line with other studies, this study found age, multimorbidity, and level of home healthcare service to be associated with OOH use.^20,21^

Older patients of non-Western ethnicity had a higher likelihood of using the OOH and had more OOH consultations when adjusting for all other parameters. The association with increased consultation frequency has been established in the total non-Western population in Denmark for OOH consultations.^22^ This is contrary to this author groups' previous research in daytime general practice showing that patients of non-Western ethnicity were less likely to use general practice, and if they did, they had fewer consultations than ethnic Danes.^23^ Whether this pattern of higher use of OOH than daytime general practice is suggestive of misuse of services, low health literacy, or communicative problems to language issues is unknown. One reason might be that patients must, in general, pay an interpreter fee themselves if needed during daytime consultations whereas interpreters are not available in OOH, which might result in lower use of daytime general practice, with patients waiting to see if symptoms worsen or not.

Increasing distance to the nearest emergency department was associated with older patients being non-users of OOH and fewer consultations if using OOH. This is in line with findings among older patients in rural Ireland, where one of the main challenges for attending OOH was difficulty accessing transportation.^24^ Likewise, the relationship between low socioeconomic status and OOH use has previously been established.^22,25^ In the current study, the association was only present for household wealth and not income. Wealth has been established as a more accurate measure of socioeconomic status than income in retirement.^26^

The 'Phone heavy' and 'High frequency' profiles showed no stable associations with user-status or frequency of consultations in OOH. One reason could be that older patients’ use of OOH are not sensitive to changes in daytime services. Research shows that older patients have the lowest number of inappropriate telephone consultations with OOH.^27^ The case may simply be that when an older patient contacts OOH they are in need of the service and cannot wait until the following workday. Further, some research points towards older patients being hesitant to contact OOH even when in need.^24^ Contrary, patients in the 'Phone and email heavy' profile were more likely to use OOH although they had a higher frequency of all daytime consultations compared with the 'Majority'.^18^ However, the profile was only observed in 1 year, which in, and of, itself was unusual because of the COVID-19 pandemic, and the sensitivity analysis showed an inverse association. Thus, no definitive conclusions can be drawn on the stability of this association.

### Implications for research and practice

This study found no sign of lower use of OOH for older patients in general practices that provided more consultations during daytime hours. Suggesting that older patients’ OOH use cannot be reduced by GPs offering more daytime consultations. The question for further research is whether a higher level of daytime consultations has an impact on older patients use of secondary health sectors (such as risk of hospital admission) or mortality. If no beneficial effects can be identified it may be more appropriate for general practice to allocate the extra resources towards other parts of the population, for example,older patients of non-Western ethnicity for whom worse outcomes may be anticipated with their inverse use of daytime versus OOH. Finally, as the findings apply only to the older population, further research is needed to explore these patterns in broader demographics.

## Data

All data are available upon reasonable request from Statistics Denmark and the Danish Healthdata Authority.
